# Data on whole genome sequencing of extrapulmonary tuberculosis clinical isolates from India

**DOI:** 10.1016/j.dib.2018.08.048

**Published:** 2018-08-24

**Authors:** Jayshree Advani, Kusum Sharma, Renu Verma, Oishi Chatterjee, Hitendra S. Solanki, Aman Sharma, Subhash Varma, Manish Modi, Pallab Ray, Megha Sharma, M.S. Dhillion, Akhilesh Pandey, Harsha Gowda, T.S. Keshava Prasad

**Affiliations:** a Institute of Bioinformatics, International Technology Park, Bangalore 560066, India; b Manipal Academy of Higher Education, Manipal, Karnataka 576104, India; c Department of Medical Microbiology, PGIMER, Chandigarh 160012, India; d School of Biotechnology, Amrita Vishwa Vidyapeetham, Kollam, Kerala 690525, India; e Center for Systems Biology and Molecular Medicine, Yenepoya Research Centre, Yenepoya (Deemed to be University), Mangalore 575018, India; f School of Biotechnology, KIIT University, Bhubaneswar, Odisha 751024, India; g Department of Internal Medicine, PGIMER, Chandigarh 160012, India; h Department of Neurology, PGIMER, Chandigarh 160012, India; i Department of Orthopedics, PGIMER, Chandigarh 160012, India; j McKusick-Nathans Institute of Genetic Medicine, Johns Hopkins University School of Medicine, Baltimore, MD 21205, USA; k Department of Biological Chemistry, Johns Hopkins University School of Medicine, Baltimore, MD 21205, USA; l Department of Pathology, Johns Hopkins University School of Medicine, Baltimore, MD 21205, USA; m Department of Oncology, Johns Hopkins University School of Medicine, Baltimore, MD 21205, USA

## Abstract

This article describes the whole genome sequencing data from 5 extrapulmonary tuberculosis clinical isolates. The whole genome sequencing was carried out on Illumina MiSeq platform to identify single nucleotide variations (SNVs) associated with drug resistance. A total of 214 SNVs in the coding and promoter regions were identified in the whole genome sequencing analysis. Among the identified SNVs, 18 SNVs were identified in genes known to be associated with first and second line drug resistance. The data is related to the research article “Whole genome sequencing of *Mycobacterium tuberculosis* isolates from extrapulmonary sites” (Sharma et al., 2017) [1].

**Specifications Table**

**Table:** 

Subject area	Biology
More specific subject area	Infectious diseases
Type of data	Raw fastq files, Excel tables and figures
How data was acquired	Illumina MiSeq
Data format	Raw and analysed data
Experimental factors	Extra pulmonary isolates from cerebrospinal fluid (CSF), joint aspirate pus and fine needle aspiration cytology were cultured on LJ slants and genomic DNA was isolated using cetyltrimethylammonium bromide (CTAB) method
Experimental features	Library preparation and sequencing was performed according to Illumina Miseq specific protocols
Data source location	Punjab and Bangalore, India
Data accessibility	Data is with this article and whole genome sequencing data is available in NCBI SRA database with accession PRJNA358480, https://www.ncbi.nlm.nih.gov/sra/?term=PRJNA358480.
	https://www.ncbi.nlm.nih.gov/sra/SRX2439868
	https://www.ncbi.nlm.nih.gov/sra/SRX2439869
	https://www.ncbi.nlm.nih.gov/sra/SRX2439870
	https://www.ncbi.nlm.nih.gov/sra/SRX2439871
	https://www.ncbi.nlm.nih.gov/sra/SRX2439872
Related research article	Whole genome sequencing of *Mycobacterium tuberculosis* isolates from extrapulmonary sites [1].

**Value of the data**

• This data provides insight into the genomic profiles of *M. tuberculosis* clinical isolates from extra pulmonary sites
• Lineage-specific SNVs identified in whole genome sequencing allows accurate strain typing and provided the information of lineage distribution of EPTB isolates
• The data also provided information on SNVs associated with conferring resistance to anti-tubercular drugs
• Since genomic profiles of EPTB isolates remains largely unexplored, this data would add value to our current knowledge on genomes of *M. tuberculosis* isolated from different infection sites

## 1 Data

**Fig. 1:**
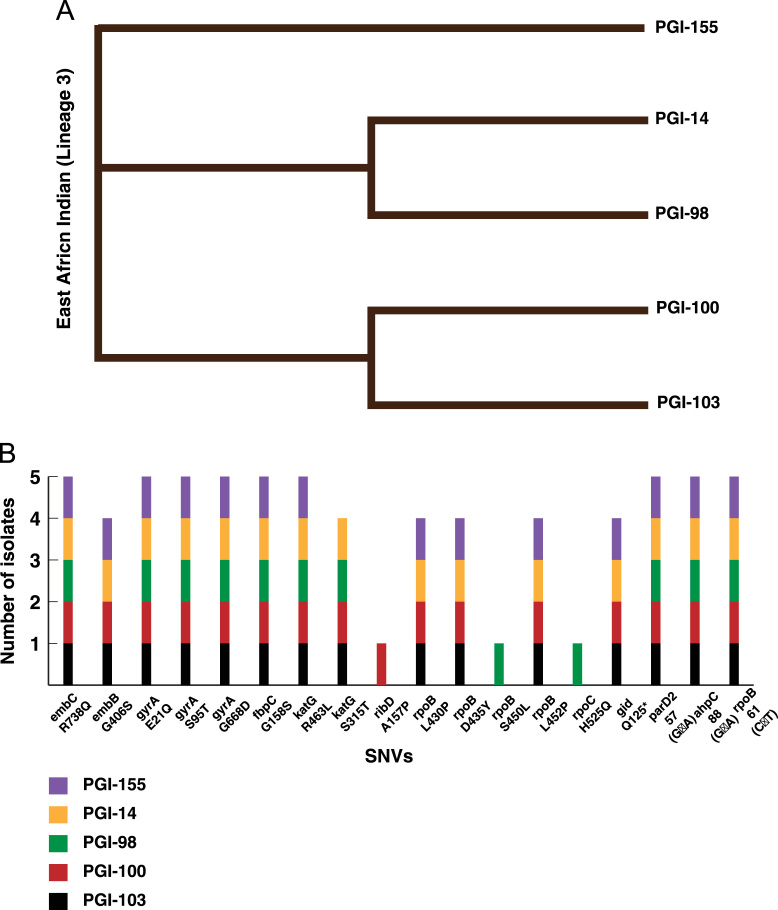
**(A)** Phylogenetic tree of five EPTB clinical isolates. **(B)** Distribution of SNVs in the coding and promoter region of genes associated with drug resistance in the five EPTB isolates.

**Fig. 2:**
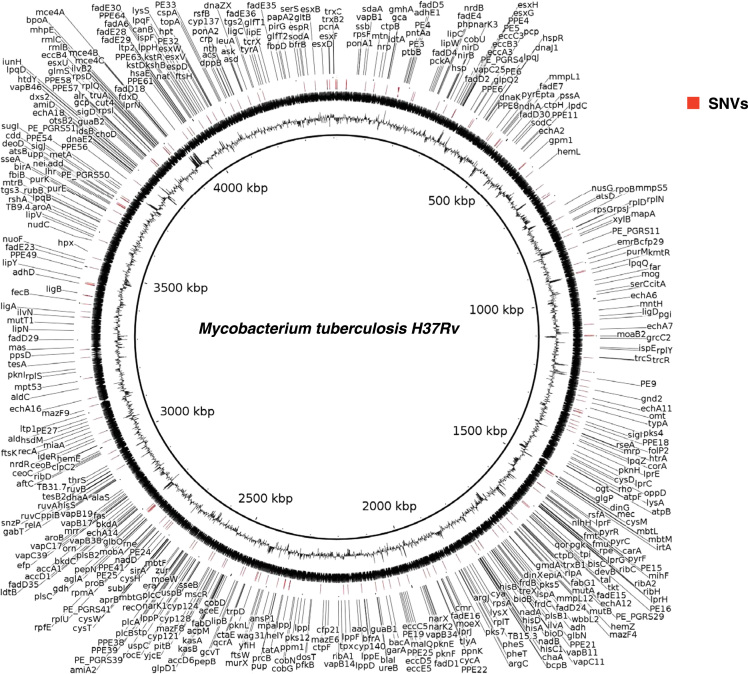
Circos plot depicting the promoter region SNVs identified in the study.

The data represents whole genome sequencing of 5 extra pulmonary isolates from 3 different sites. All five clinical isolates sequenced in this data set belonged to East-African-Indian lineage (Lineage 3) (Fig. 1A). A scientific interpretation of this data set was performed by Sharma et al. [1]. Data analysis led to the identification of 15 SNVs in the coding region of genes (Fig. 1B), which are known to confer drug resistance to first and second line anti-tubercular drugs (Supplementary Table 1A). Apart from known drug resistance SNVs, we also identified 199 SNVs in the promoter regions corresponding to 157 genes (Supplementary Table 1B**) (**Fig. 2**).** Three of these 157 genes are associated with drug resistance show promoter region SNVs in all of the 5 isolates (Fig. 1B).

## 2 Experimental design, materials and methods

### 2.1 Culturing and DNA isolation of extrapulmonary isolates

The 5 EPTB isolates were obtained from Department of Medical Microbiology, The Postgraduate Institute of Medical Education and Research, Chandigarh, India. The isolates were cultured and maintained as described in [1]. The LJ slants were incubated at 37 °C for a maximum period of 8 weeks. They were inspected daily for growth or for contamination. The isolates were then tested to rule out non tuberculous mycobacteria (NTM) or other infection and were cultured for DNA extraction as previously described [1]. DNA was extracted from the isolates cultured on the LJ slants using cetyltrimethylammonium bromide (CTAB) protocol [2].

### 2.2 Library preparation and sequencing

DNA libraries were constructed and sequencing was carried out on Illumina MiSeq instrument as described previously [1]. Sequencing was performed using a 2 ×100 paired-end (PE) configuration (Table 1).

**Table 1:** Raw data statistics.

Platform				
Illumina MiSeq (2*100) paired end				
**Sample ID**	**Category**	**R1**	**R2**	**Total Reads**
PGI-14	Cerebrospinal fluid(CSF)	2,532,274	2,532,274	5,064,548
PGI-98	Joint aspirate pus	2,250,203	2,250,203	4,500,406
PGI-100	Fine needle aspiration cytology (cervical lymph node)	2,088,387	2,088,387	4,176,774
PGI-103	Fine needle aspiration cytology (cervical lymph node)	2,315,946	2,315,946	4,631,892
PGI-155	Fine needle aspiration cytology (cervical lymph node)	2,454,773	2,454,773	4,909,546

### 2.3 Variant calling and data analysis

Paired end reads were quality checked using FastQC version-0.11.5. Raw reads of Phred quality score of < 20 were discarded. High quality reads were mapped to the H37Rv reference genome (NC_000962.3) using Burrows-Wheeler Alignment Tool (BWA version-0.7.15) [3]. Variants were identified using GATK [4]. The variants were annotated using in-house perl scripts. Phylogenetic analysis was carried out using KvarQ version-0.12.2 [5]. SNVs identified in the isolates were used to generate phylogenetic tree FastTree version-2.1.10 [6].

## Appendix A Supplementary material

**Supplementary Table 1A:** List of SNVs known to confer resistance to first and second line drugs. **Supplementary Table 1B**: List of SNVs identified in the promoter region. Supplementary material
