# Supplementary material for: Data on whole genome sequencing of extrapulmonary tuberculosis clinical isolates from India
Source: Data Brief. 2018 Aug 24;20:617–22. doi: 10.1016/j.dib.2018.08.048 (PMC6127979; doi:10.1016/j.dib.2018.08.048)
Supplement: Supplementary file 1 — Supplementary material [file mmc1.docx]

Conflict of Interest

All author confirms no conflict of interest.
